# The six minute walk test accurately estimates mean peak oxygen uptake

**DOI:** 10.1186/1471-2466-10-31

**Published:** 2010-05-26

**Authors:** Robert M Ross, Jayasimha N Murthy, Istvan D Wollak, Andrew S Jackson

**Affiliations:** 1 Baylor College of Medicine, 3333 Richmond Ave, 2nd Floor, Houston, Texas 77098, USA; 2 Division of Pulmonary Critical Care and Sleep Medicine, University of Texas Health Science Center at Houston, Texas, USA; 3 Memphis Lung Physicians, 6025 Walnut Grove, Memphis, TN 38120, USA; 4 Department of Health and Human Performance, University of Houston, Houston, Texas, Adjunct Professor of Medicine, Baylor College of Medicine, Houston, Texas, USA

## Abstract

**Background:**

Both Peak Oxygen Uptake (peak VO2), from cardiopulmonary exercise testing (CPET) and the distance walked during a Six-Minute Walk Test (6 MWD) are used for following the natural history of various diseases, timing of procedures such as transplantation and for assessing the response to therapeutic interventions. However, their relationship has not been clearly defined.

**Methods:**

We determined the ability of 6 MWD to predict peak VO2 using data points from 1,083 patients with diverse cardiopulmonary disorders. The patient data came from a study we performed and 10 separate studies where we were able to electronically convert published scattergrams to bivariate points. Using Linear Mixed Model analysis (LMM), we determined what effect factors such as disease entity and different inter-site testing protocols contributed to the magnitude of the standard error of estimate (SEE).

**Results:**

The LMM analysis found that only 0.16 ml/kg/min or about 4% of the SEE was due to all of the inter-site testing differences. The major source of error is the inherent variability related to the two tests. Therefore, we were able to create a generalized equation that can be used to predict peak VO2 among patients with different diseases, who have undergone various exercise protocols, with minimal loss of accuracy. Although 6 MWD and peak VO2 are significantly correlated, the SEE is unacceptably large for clinical usefulness in an individual patient. For the data as a whole it is 3.82 ml/kg/min or 26.7% of mean peak VO2. Conversely, the SEE for predicting the mean peak VO2 from mean 6 MWD for the 11 study groups is only 1.1 ml/kg/min.

**Conclusions:**

A generalized equation can be used to predict peak VO2 from 6 MWD. Unfortunately, like other prediction equations, it is of limited usefulness for individual patients. However, the generalized equation can be used to accurately estimate mean peak VO2 from mean 6 MWD, among groups of patients with diverse diseases without the need for cardiopulmonary exercise testing. The equation is:

## Background

The Six-Minute Walk Test (6 MWT) is an inexpensive, relatively quick, safe and a well-tolerated method of assessing the functional exercise capacity of patients with moderate-to-severe heart or lung disease. Its use has found popularity in following the natural history of various diseases, for timing of procedures such as heart or lung transplantation and for measuring the response to medical interventions [1].

Cardiopulmonary Exercise Testing (CPET) with the measurement of peak oxygen uptake (peak VO2) is the "gold standard" for assessing aerobic capacity. However, the test is relatively expensive and time consuming. Although CPET may be used periodically during a study, generally the 6 MWT is used for the routine following of study patients' exercise capacity.

There has been a substantial body of literature published looking at the relationship between 6 MWT and peak VO2 in individuals [2-11]. These studies have found that the standard error of estimate (SEE) in the correlation equation between 6 MWD and peak VO2 is quite large. However, the source of this large error has not been explored. Further, the relationship between the mean peak VO2 and the mean Six Minute Walk Distance (6 MWD) among different study groups, has not been assessed. This could have significant value when comparing study groups in terms of average peak VO2 when only 6 MWD data is available. For example, if a therapeutic intervention showed promise in one study but not another, a potential reason could be that the groups had significantly different mean peak VO2's and the therapy is only efficacious for those with adequate aerobic reserve.

A potential problem in deriving an equation to estimate mean peak VO2 from mean 6 MWD is that the two tests are not performed uniformly at different institutions throughout the world. Type of disease and test administration factors could significantly influence the relationship. These include the manner in which the 6 MWT is performed, whether there is a learning 6 MWT performed first, the CPET protocol used, the test mode used, treadmill or cycle ergometer, and whether the individual uses supplemental oxygen for the 6 MWT. All of these factors are potential sources of confounding bias that could conceivably make a generalized equation of limited practical use. However, the magnitude of these variables on the SEE has not been explored.

This study was designed to examine the relationship between 6 MWD and peak VO2 in diverse groups of patients with various cardiac, circulatory and pulmonary disorders, who were tested under different clinical protocols, to determine if a useful generalized equation to estimate peak VO2 from 6 MWD could be derived.

## Methods

The data for this study came from two sources. The first was data from a sequential, retrospective chart analysis of 50 patients who had completed both a CPET and 6 MWT on the same day performed at our institution, The Methodist Hospital, Baylor College of Medicine in Houston, Texas. Many of these patients had both studies as part of a heart or lung transplant program. The authors were not involved in the patient selection or decision to have the tests. The study was approved by The Methodist Hospital Research Institute Office of Research Protections. If the same patient had more than one test, only the first test was used for analysis. A total of 48 patients met these criteria. The patients had a diverse group of cardiopulmonary disorders including pulmonary hypertension, interstitial lung disease and chronic obstructive pulmonary disease. There were 25 women with a mean age of 55.4 ± 10.1 years and 23 men with a mean age of 53.3 ± 13.1 years.

The 6 MWT was performed walking a corridor of 100 feet in length utilizing the protocol outlined by the American Thoracic Society ATS [1]. Some of our study patients required supplemental oxygen while performing the test, however, all completed the 6 MWT. Peak oxygen uptake was obtained from expired gas analysis using an Eric Jaeger ™ Oxycon Alpha or Oxycon Pro. The patients were encouraged to exercise to voluntary exhaustion. The CPET protocol was designed by an experienced technician so that each patient would reach maximum power output by approximately ten minutes. The women had a mean 6 MWD of 346.6 ± 128.5 meters and mean peak VO2 of 12.5 ± 3.1 ml/kg/min. The men had a mean 6 MWD of 361.3 ± 136.3 meters and mean peak VO2 of 13.7 ± 3.5 ml/kg/min. Their pooled results and linear regression statistics are shown in Table 1.

**Table 1:** Sample and linear regression characteristics for all subjects contrasted by study.

Study	Sample Characteristics				Linear Regression Statistics				
	
	Disease	n	peak VO2 Mean ± SD	6 MWT Mean ± SD	Slope	Intercept	R	SEE	(SEE/Mean) × 100
Cahalin 1996 (3)	CHF	45	12.4 ± 4.5	310 ± 103	0.028	3.583	0.65	3.44	27.7

Cahalin 1995 (2)	ESLD	60	9.6 ± 3.8	294 ± 139	0.019	4.042	0.69	2.81	29.2

Lucas 1999 (6)	CHF	307	14.2 ± 4.9	391 ± 105	0.027	3.666	0.59	3.99	28.1

Miyamoto 2000 (7)	PH	27	13.9 ± 4.4	377 ± 115	0.026	4.213	0.68	3.25	23.4

Opasich 2001 (8)	CHF	269	14.5 ± 4.9	378 ± 95	0.027	4.498	0.59	3.42	23.6

Roul 1998 (9)	CHF	114	16.8 ± 4.5	437 ± 108	0.009	12.910	0.21	4.43	26.4

Starobin 2006 (10)	COPD	49	14.0 ± 4.4	436 ± 89	0.027	2.184	0.55	3.69	26.4

Zugck 2000 (11)	DC	112	15.6 ± 5.2	463 ± 107	0.033	0.113	0.69	3.78	24.2

Faggiano 1997 (4)	CHF	26	15.1 ± 3.9	419 ± 121	0.019	7.260	0.58	3.25	20.8

Lipkin 1986 (5)	CHF	26	14.0 ± 4.1	452 ± 147	0.019	5.271	0.70	2.95	21.0

Baylor	CPD	48	13.1 ± 3.4	354 ± 131	0.017	6.921	0.68	2.50	19.1

All Data	All	1,083	14.3 ± 4.8	393 ± 115	0.025	4.682	0.59	3.82	26.7

ESLD - End Stage Lung Disease; DC - Dilated Cardiomyopathy; CHF - Congestive Heart Failure; PH -Pulmonary Hypertension; COPD - Chronic Obstructive Pulmonary Disease; CPD - Various Cardiopulmonary Disorders. Peak VO2 - peak oxygen uptake (ml/kg/min.); 6 MWD- distance walked (meters) during the 6-minute walk test.

We also performed a literature search up through mid 2006 utilizing Pub Med. We looked for studies where raw data displaying the relationship between 6 MWD and peak VO2 was presented. 10 studies [2-11] were found. Eight of the studies published the data only as bivariate scattergrams. In these cases, the graphs from these articles were electronically copied to a program where the coordinates of each point could be ascertained. These values were then multiplied by appropriate scaling factors to obtain each individual's peak VO2 and 6 MWD values. Points of some subjects were superimposed on each other making it impossible to recover all the data. However, we were able to obtain 95% of all published data points. These studies were performed at sites around the world, including the US, Europe and Japan. They encompassed patients with many different heart and lung disorders, exercised under various protocols. Table 2 lists information regarding the CPET and 6 MWT protocols utilized by the different studies. These studies, each of uniform patient diseases and exercise protocols, were used for comparison to the results from our study group. We found that the correlation coefficient and SEE of our data were similar to those from these other studies even though our group consisted of patients with a mixture of cardiopulmonary disorders exercised according to our protocol. This suggested that different patient diseases as well as different CPET and 6 MWT techniques (which we will call collectively the "inter-site effect") might not be major factors in the size of the SEE.

**Table 2:** Exercise characteristics of the different studies

	CPET				6 MWT		
**Author**	**Type**	**Stop**	**AT**	**RER**	**Length**	**Practice**	**Stop**

Cahalin (2)	cycle	Pt	70%	NR	168 ft	yes	Distress, O2 < 80% *

Cahalin (3)	Cycle	Pt	88%	NR	168 ft	Yes	Distress

Faggiano (4)	Cycle	Pt	88%	NR	NR	Yes	NR

Lipkin (5)	TM	Pt	NR	NR	20 M	Yes	NR

Lucas (6)	Cycle	Pt	NR	NR	20 M	Yes?	NR

Myamoto (7)	Cycle	NR	NR	NR	NR	NR	NR

Opasich (8)	Cycle	NR	82%	NR	34 M	Yes	Distress

Roul (9)	Cycle	NR	100%	NR	Ped	NR	NR

Starobin (10)	Cycle	NR	NR	NR	Cor	NR	NR

Zugck (11)	Cycle	Pt	NR	NR	132 M	NR	None

Baylor	Cycle TM	Pt	NR	NR	100 ft	NR	None *

CPET- Cardiopulmonary Exercise Test: Type: cycle- cycle ergometer or treadmill (TM) using various protocols of gradually increasing power output. Stop: Pt: voluntary exhaustion. AT: % of group that had an anaerobic threshold. RER: Average maximum respiratory exchange ratio. 6 MWT- Six Minute Walk Test: Length: length of corridor in feet or Meters. Ped: pedometer, Cor: corridor but length not reported. Stop (Criteria for terminating walk): Distress: various distress criteria, O2 < 80%: O2 saturation dropping below 80%, None: Everyone completed test. NR: not reported, * some patients used O2 during the walk.

To study the magnitude of this "inter-site effect" on the SEE more rigorously, we used Linear Mixed Models regression analysis (LMM). In this regard, the inter-site effect encompassed the various differences in disease extent and type, as well as exercise protocols and other variability among the different data sets obtained from the different studies. For this analysis, each of the studies was treated as a random variable. Both random intercept and random coefficient models were examined [12,13]. A log ratio test [14] was used to determine which model fit the data better. The method of obtaining estimates of the unknown parameters of the LMM was by optimizing a likelihood function. STATA 9.0 [14] was used for all analyses.

## Results

### Scanned Data

Of the 10 studies found in the literature, the data from eight were obtained from scans of the published scattergrams. To our knowledge, this technique has not been used before. In order to validate it, we compared statistics derived from our "measured" data from the graphs to values published in the articles. The largest difference between the mean 6 MWD reported and calculated from measured data was only 18 meters. This was for the sample with the most under represented data points [8]. The largest mean difference for the remaining samples was just 4 meters. The largest difference in the standard deviations between reported and graphed data was only 3 meters. For peak VO2 the largest difference was 0.5 ml/kg/min [10] and the next was 0.2 ml/kg/min. The largest difference in the standard deviations was 0.5 ml/kg/min while the next highest was 0.3 ml/kg/min. The largest difference in the correlations reported and those that we obtained from the scanned data was only 0.06. These findings indicate that the data obtained from scans of the published scattergrams were accurate as they provided an excellent fit of the published results.

### Linear Regression Analysis

Table 1 provides sample characteristics for each of the 11 studies and all studies combined. For the Baylor group, the mean peak VO2 was 13.1 ml/kg/min (± 3.4 ml/kg/min). The mean 6 MWD was 354 (± 131) meters. The correlation between peak VO2 and 6 MWD was 0.68 (p < 0.001) with a SEE of 2.50 ml/kg/min. The sample sizes of the studies from the literature ranged from 26 to 307 patients. The mean peak VO2 of the groups ranged from 9.6 to 16.8 ml/kg/min, while the range for the 6 MWD means was from 294 to 463 meters. Table 1 also provides linear regression statistics. While all correlations were statistically significant, they ranged from a low of 0.21 to a high of 0.70. Standard errors of estimate ranged from a low of 2.50 to 4.43 ml/kg/min. The SEE normalized by mean peak VO2, ranged from 19.1 to 29.2%. The correlation for all 1,083 patients combined was 0.59 and the SEE was 3.82, nearly 27% of the mean of 14.3 ml/kg/min.

**Figure 1:**
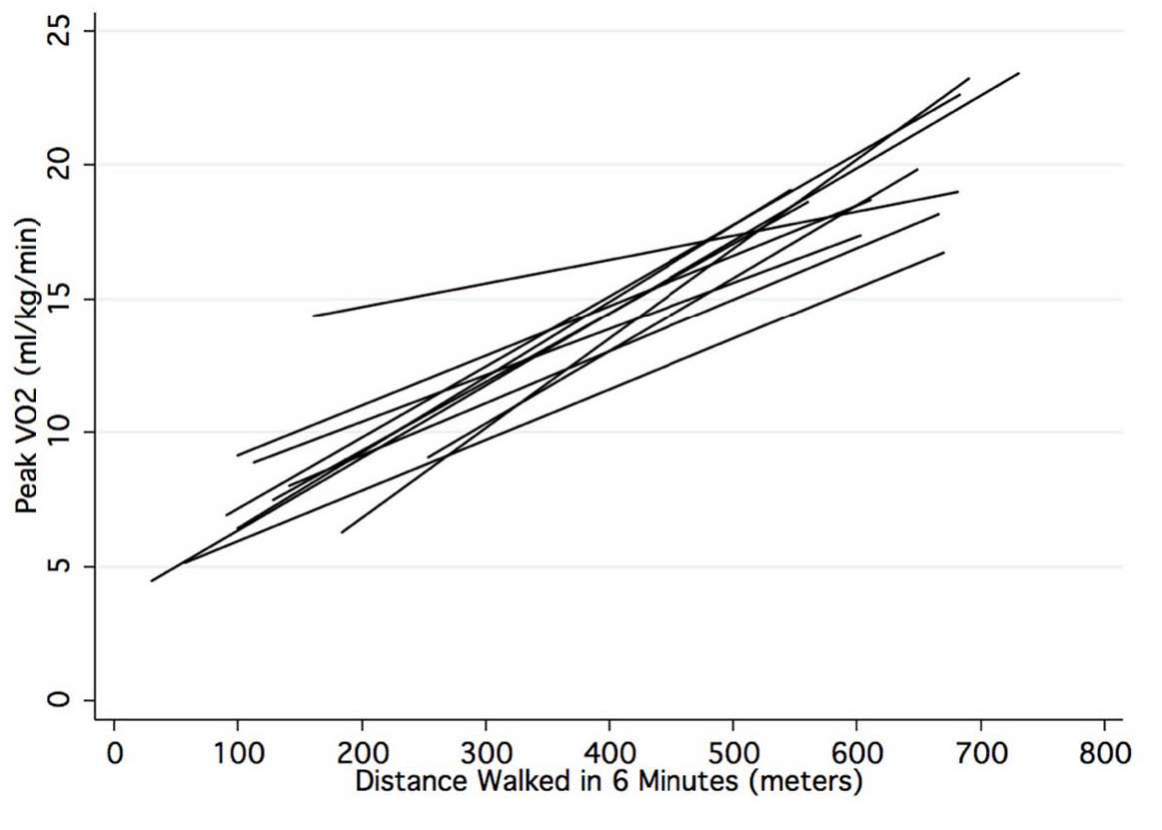
**Linear regression lines defining the relation between the distance walked in 6 minutes and peak VO2 for the 11 studies**.

Figure 1 gives the regression lines for estimating peak VO2 from 6 MWD for each of the 11 studies. The slopes ranged from 0.017 to 0.33 and the intercept range was from 0.113 to 12.9. The linear regression equation derived from the combined data of this diverse group of 1,083 patients who had their 6 MWT and CPET performed under various different protocols had a slope of 0.025 and intercept of 4.682.

### Linear Mixed Model Analysis (LMM)

Table 3 gives the LMM analysis. Provided are two models, random intercept (LMM-I) and random coefficient (LMM-II) [12]. The coefficients for the intercepts and 6 MWD slope for the fixed part of the two models were significantly different from zero (p < 0.001). The equations of the two models were nearly identical, with a difference of just 0.322 for intercepts and 0.001 for 6 MWD slope. The fixed-effect LMM SEE, which represents population-averaged measurement error estimates [13], and the linear regression SEE for the entire group were identical at 3.82 ml/kg/min.

**Table 3:** Maximum likelihood estimates for the linear mixed model to estimate peak VO2 from 6-minute walk distance.

Parameter	M-I: Random Intercept Estimate ± SEE	M-II: Random Coefficient Estimate ± SEE
Fixed Component		

Intercept	4.626 ± 0.516	4.948 ± 0.990

6-Minute Walk	0.024 ± 0.001	0.023 ± 0.002

SEE_(AVG)_	3.82	3.82

Random Component		

Intercept_(SD)_	0.91 ± 0.25	2.80 ± 0.79

Slope_(SD)_		0.01 ± >0.01

R_(Slope, Intercept)_		-0.94 ± 0.05

SEE_(CON)_	3.73 ± 0.08	3.66 ± 0.08

Log likelihood	-2972.51	-2964.69

The log ratio test [14] found that the random coefficient model (LMM-II) provided a better fit than the random intercept model (Chi square = 15.65, p = 0.0004) documenting that the differences in the regression slopes and intercepts graphed in Figure 1 were due to inter site differences and not chance variation. The correlation for the slopes and intercepts of the 11 studies was -0.94 demonstrating that the steeper slopes among the 11 studies were associated with lower intercepts. The SEE of the random effects model was 3.66 ml/kg/min (95% CI, 3.54 to 3.86). This SEE is lower than for the fixed effects model because the site-specific variation in slopes and intercepts is statistically controlled, yielding an estimate of the 6 MWT prediction accuracy free of any inter-site effect. This SEE was only 0.16 ml/kg/min lower than the fixed-effect SEE of 3.82, indicating that the inter-site effects were small and accounted for only 4% of the overall SEE.

**Figure 2:**
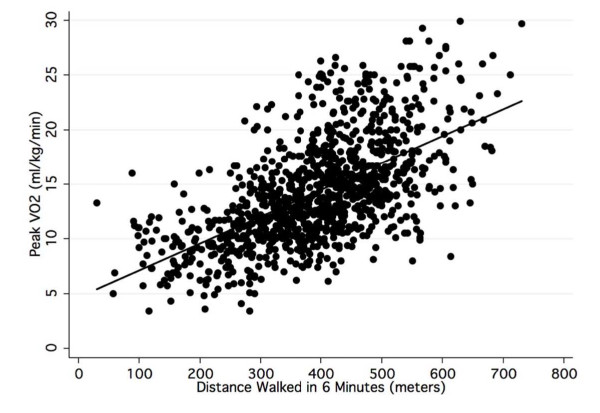
**Scattergram and linear regression line for the distance walked in 6 minutes and peak VO2 for all patients from the 11 studies**.

Figure 2 is a bivariate scattergram of all the patient data and the regression line (LMM II) to estimate peak VO2 from 6 MWD. The scattergram illustrates that there is considerable variability in peak VO2 for any given 6 MWD.

**Figure 3:**
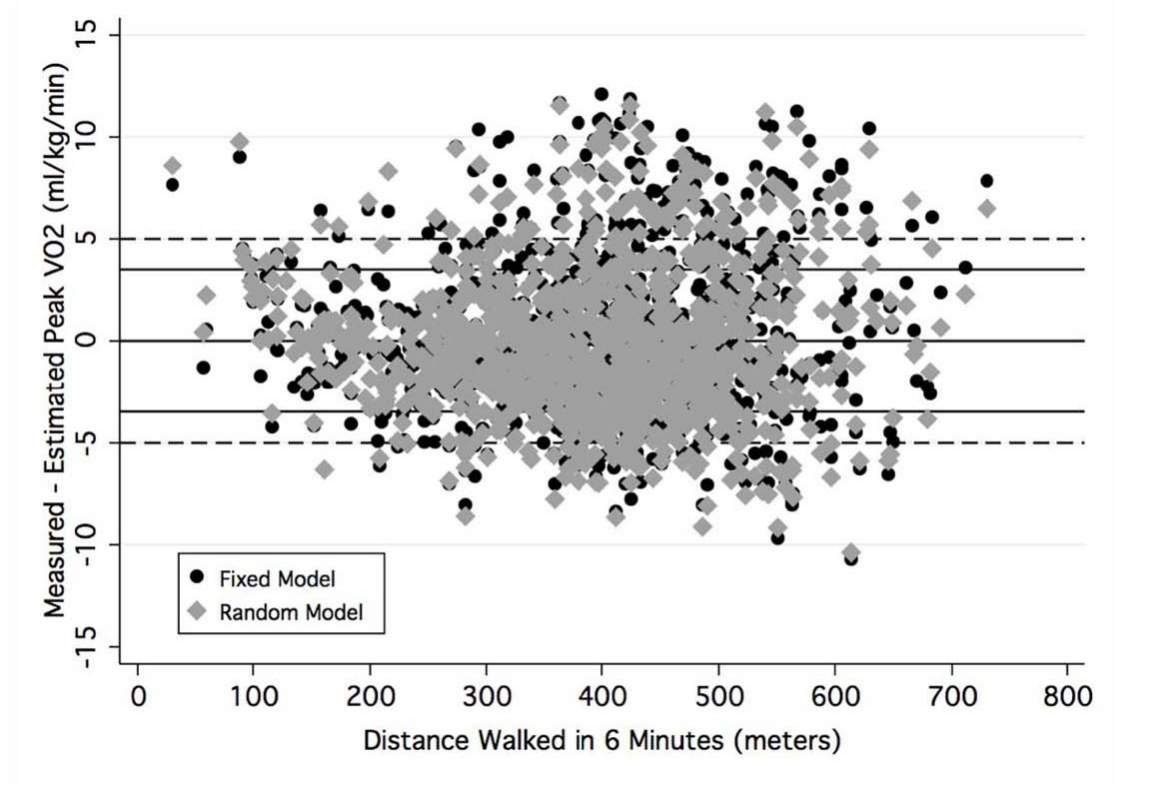
**Bivariate plot of the residuals of the LMM fixed and random models contrasted by distance walked in 6 minutes**.

Figure 3 graphically presents the bivariate scattergram of 6 MWD by the LMM II residuals for the fixed (black circle) and random (gray triangle) models. The random model residuals controlled for variation among test sites. Provided for reference are solid lines for residuals of 0 and ± 3.5 ml/kg/min. The dashed lines represent prediction errors ± 5 ml/kg/min. Analysis of the distributions of residuals showed that 67% of the fixed equation residuals were ± 3.5 ml/kg/min and 82% were ± 5 ml/kg/min. As Figure 3 documents, the difference in residuals between LMM II fixed and random models was small and not systematic. An analysis of the LMM II random equation residuals showed that 68% of the errors were ± 3.5 ml/kg/min and 83% were ± 5 ml/kg/min.

### Estimation of mean Peak VO2

The data in Table 4 examines the accuracy of the generalized equation (LMM II) to estimate the mean peak VO2 from the mean 6 MWD for each of the 11 studies. This analysis shows that the range for the mean differences, between measured and estimated peak VO2, is -2.1 to 1.8 ml/kg/min. The standard deviation of the mean error estimates is 1.1 ml/kg/min, or only 7.7% when normalized by mean peak VO2.

**Table 4:** Accuracy of the generalized LMM equation in estimating mean measured peak VO2 from mean 6 MWD.

Study	M-peak VO2	6 MWD	E-peak VO2	Measured - Estimated
Cahalin (3)	12.4	310	12.1	0.3

Cahalin (2)	9.6	294	11.7	-2.1

Lucas (6)	14.2	391	13.9	0.3

Miyamoto (7)	13.9	377	13.6	0.3

Opasich (8)	14.5	378	13.6	0.9

Roul (9)	16.8	437	15.0	1.8

Starobin (10)	14.0	436	15.0	-1.0

Zugck (11)	15.6	463	15.6	0.0

Faggiano (4)	15.1	419	14.6	0.5

Lipkin (5)	14.0	452	15.3	-1.3

Baylor	13.1	354	13.1	0.0

Grand Mean	13.9	392	13.9	0.0

M-peak VO2 (Measured mean peak VO2 from CPET)

E-peak VO2 (Estimated mean peak VO2 from 6 MWD):

Mean peak VO2 = 4.948 + (0.023 × 6 MWD) (SEE 1.1 ml/kg/min)

## Discussion

The results of our study, all other individual studies and all data combined showed that 6 MWD and peak VO2 were significantly correlated. Although, the site-specific prediction equations, which are presented in Table 1, differed somewhat, they all had large SEE's, particularly as a percent of mean peak VO2. LMM analysis showed that inter-site variability such as disease type and different testing protocols did not substantially increase the SEE. The LMM II error estimates for the fixed (3.82 ml/kg/min) and random models (3.66 ml/kg/min), although statistically significantly different, were almost identical. The fixed effects SEE of 3.82 ml/kg/min is the average error estimate of all sites [15]. The random effects SEE of 3.66 ml/kg/min is the SEE statistically excluding all factors associated with variability among the sites and study groups by utilizing empirical Bayes predictor, or the best linear unbiased predictor [12], which accounts for the variability among test sites. The degree of error that is due to differences in test site variation was just 0.16 ml/kg/min or 4% of the SEE. The analysis of the residuals in Figure 3 documents this small difference in measurement error. This finding indicates that a generalized equation can be used to estimate peak VO2 from 6 MWD with little loss of accuracy. Generalized across the 11 test sites, the SEE was 3.82 ml/kg/min, which was about 27% of mean peak VO2.

Intuitively, it might be thought that inter-site factors would have a larger effect on the SEE. Most of the authors from the 10 additional studies we evaluated used patients with a uniform disorder and exercised them in a uniform manner in an attempt to minimize any error introduced by these factors. However, our study found that these factors were a minor source of error. We believe this is because the major source of error is the random, inherent, within-subject measurement errors associated with CPET and the 6 MWT. In this regard, although the test-retest reliability for peak VO2 from CPET for normal people has been found to be about 0.96, this represents a standard error of measurement of about 2 ml/kg/min [16]. Similar results have been found for patients. A group of patients with fibrotic interstitial pneumonia had a coefficient-of-variation of 10.5% of peak VO2. This represented approximately 2 to 2.5 ml/kg/min [17]. This value is about 15% of the mean peak VO2 of the pooled patient data we obtained.

The 6 MWT also has significant inherent variability. In one study, the within-subject variability for 6 MWD was 4.2% or about ± 34 m [17]. In another study, after an initial learning period, patients with chronic heart or lung disease had a within-person standard deviation for 6 MWD of about 6%. This represented a 95% likelihood of about ± 40 m [18]. Since the vast majority of the patients evaluated in the studies we reviewed had a 6 MWD between 200 and 600 m, this could lead to as much as 20% variability in 6 MWD. Based on the correlation equation we obtained, this could contribute an additional error in estimating peak VO2 of about +/- 1 ml/kg/min.

Although walking is an aerobic activity and, for people with significant aerobic limitation, may be a maximal exercise activity, there are many reasons why people with a similar peak VO2 might have a different 6 MWD. The 6 MWT is a voluntary effort where the person's walking speed can vary and the person might even stop and rest for a period of time. Two people with the same peak VO2 might choose different walking strategies. One might walk more slowly, the other faster but rest periodically. Patients might chose different average walking speeds based on physiological factors such as work-of-breathing, auto-PEEP [19], work-of-the-heart or how much carbon dioxide retention the individual can comfortably tolerate. For example, in a group of patients with congestive heart failure, the VO_2 _measured at the end of the 6 MWT was on average 15% lower than peak VO2. However, it was equal to or higher than measured peak VO2 from CPET in about 25% of the patients [4]. Psychological factors such as anxiety or a patient's unique perception of pain, dyspnea or discomfort due to their abnormal physiology also can affect 6 MWD. In this regard encouragement has been shown to increase 6 MWD in sick patients [20].

If the test-retest variations are random for both peak VO2 and 6 MWD measurements, as would be expected, it is not surprising that we obtained a SEE of 3.66 ml/kg/min independent of the error introduced by site differences. It is unlikely that utilizing a different walk time would improve predictive accuracy, as the physiological principles are the same. Similar correlations and standard errors have been found utilizing the two and the twelve-minute walk tests [21,22] as well as for predicting peak VO2 from maximal exercise treadmill time [23].

It is possible that part of the large SEE may not have come from the intrinsic variability of the testing techniques but from improper patient selection. The physiologic basis behind utilizing the 6 MWT to estimate peak VO2 is that maximal exercise tests correlate quite well with peak VO2 [24]. However, the 6 MWT is a submaximal exercise test for most people with normal or mild-to-moderately reduced aerobic capacity. Submaximal exercise tests require some estimate of internal effort, such as exercise heart rate so that maximal exercise capacity can be predicted, before they can be used to adequately estimate peak VO2. That is, if the 6 MWT is a submaximal effort, there is no physiologic basis for a close correlation between maximal walking speed and peak VO2. Therefore, it is possible that including people whose 6 MWD was not limited by aerobic factors might have affected the size of the SEE we found. In normal people, the distance walked in 6 minutes of voluntary non-markedly-encouraged walking does not vary due to peak VO2 but to other factors such as gait limitation [25]. Normative values for the 6 MWT have been published [26]. For a 60 year old man, the lower limit of normal would be about 450 m. To exclude the effect that people with a potentially normal aerobic capacity might have on the SEE, we analyzed the combined data using only subjects who walked <450 m. There were 742 patients in this group. The mean peak VO2 was 12.9 ml/kg/min and the SEE for this group was +/- 3.44 ml/kg/min or 26.7% of the mean. Thus, even when patients with potentially normal functional capacity are excluded, the accuracy for estimating an individual's peak VO2 is poor. This indicates that the large SEE that we observed was not due to improper patient selection.

Although the SEE is 3.82 ml/kg/min when predicting an individual's peak VO2, it is only 1.1 ml/kg/min when predicting the mean peak VO2 of a study group (Table 4). This much smaller SEE also indicates that inter-site factors are not as important as the intrinsic variability of the test results. Further, this finding suggests the variability is random because with larger numbers, random effects would tend to cancel out and the SEE would be smaller. This is what we found.

A meta analysis is used to combine data from several studies to expand generalizability. The common method is to use the means of published results weighted by sample size. This is the first instance, to our knowledge, to reproduce data at the individual level from scanned scattergrams. Our comparative analyses of the scanned and published data documented that the errors were small and likely random. The major advantage of this approach is that we cannot only examine mean differences, but more importantly, estimate individual variation. This is shown by comparing the results provided in Figures 2 and 3 and the data in Table 4. The error analysis in Figure 3 showed that 33% of individual differences between measured and peak VO2 estimated with the fixed effect equation (LMM II) were greater than ± 3.5 ml/kg/min and nearly 20% were greater than ± 5 ml/kg/min. In contrast, the data in Table 4 shows that when the level of analysis was the average value, the prediction error was quite small, varying from -2.1 to 1.8 ml/kg/min. The correlation between peak VO2 and 6 MWD for all 1,083 individual patient data was 0.59. The correlation between peak VO2 and 6 MWD for the means of the 11 data sets was 0.82.

Our findings suggest that 6 MWD has too large of a SEE to be clinically useful for estimating peak VO2 for an individual. However, we were able to obtain only 6 MWD and peak VO2 for each data point. It is possible that with more information, and utilizing multiple linear regression analysis, a more accurate equation could have been derived. Several studies have utilized multiple linear regression analysis for patients with uniform diseases exercised with uniform protocols and still had relatively large standard errors [2,3,27]. Therefore, we doubt that this would dramatically improve accuracy.

Although the 6 MWT does not accurately predict an individual's peak VO2, many investigators have found it useful for therapeutic decision-making in moderate-to-severely ill patients [1]. 6 MWD has also been found to correlate reasonably well with the New York Heart Association (NYHA) lower functional classes [28]. The 6 MWT may act as a somewhat more objective, expanded NYHA scale [25] which could potentially allow researchers to monitor more subtle changes in exercise capacity in an individual or in a group. In this regard, serial exercise testing over about one year revealed that changes in peak VO2 were directly proportional to changes in 6 MWD [11].

Presently, no equation has been published that allows estimation of mean peak VO2 from mean 6 MWD across a large spectrum of patient groups with different diseases and exercise protocols. Our study provides this equation. Its accuracy is similar to population specific equations. However, when using this equation researchers should be cautious to exclude individuals whose 6 MWD is not limited by aerobic factors as this could lead to large errors. Maximum walking speed is generally less than 4-4.5 mph or about 700 m. in six minutes. Only 5 people in the studies we reviewed had a 6 MWD > 700 m. Based on the equation we derived, a 6 MWD of 700 m would predict a peak VO2 of about 21 ml/kg/min. In one of the studies we reviewed, the peak VO2 and 6 MWD of 10 normal subjects were reported [5]. Peak VO2 ranged from 26 to 35 ml/kg/min. The corresponding 6 MWD for these two subjects, at the extremes, were 666 m and 700 m, respectively. Our predictive equation would estimate a peak VO2 of 21.3 and 22.2 ml/kg/min. That is, utilizing the equation we derived, 6 MWD poorly predicts, and substantially underestimates, peak VO2 in people with a relatively normal aerobic capacity. Thus, people with a peak VO2 above about 20 ml/kg/min should be excluded from the group, when utilizing the equation we derived, to estimate mean peak VO2. If an individual's peak VO2 is not known, we recommend utilizing the equation only for people with moderate-to-severe heart or lung disease, excluding people whose 6 MWD is above 600 meters, as few of the patients we evaluated walked further than this and this value would estimate a peak VO2 of about 20 ml/kg/min.

## Conclusions

Based on data from 1,083 patients, we found a SEE of 3.82 ml/kg/min when predicting an individual's peak VO2 from 6 MWD. Utilizing LMM analysis we found inter-site differences contributed little to the size of this value so the equation can be generalized and used for patients with various diseases exercised under various protocols. However, the large SEE suggests poor prediction accuracy for clinical purposes when assessing an individual.

Conversely, for groups of patients with moderate-to-severe heart or lung disease, the generalized equation quite accurately estimated mean peak VO2 from mean 6 MWD. The equation is:

If individuals with normal aerobic capacity or a 6 MWD over about 600 meters are excluded, this equation can be used to compare the average peak aerobic capacity of different study groups even if they have different diseases and have exercised under slightly different 6 MWT protocols. We believe this equation could be useful for comparing study groups, in terms of average peak aerobic capacity without the need for CPET, where the 6 MWT is used to monitor the natural history of a disease or determine the efficacy of various forms of treatment.

## Competing interests

The authors declare that they have no competing interests.

## Authors' contributions

RMR was responsible for the concept and design of the study, supervision of data acquisition, was a significant manuscript writer and is the corresponding author. JNM was responsible for data acquisition and literature review. IDW was responsible for data acquisition and literature review. ASJ was responsible for statistical expertise, data analysis and interpretation, and was a significant manuscript writer. All authors read and approved the final manuscript.

## Pre-publication history

The pre-publication history for this paper can be accessed here:

http://www.biomedcentral.com/1471-2466/10/31/prepub
